# First Description of Bilateral Lung Transplantation in Patient with Birt–Hogg–Dubé Syndrome: A Case Report and Surgical Feasibility Insight

**DOI:** 10.3390/life15121814

**Published:** 2025-11-27

**Authors:** Tomasz Stącel, Jakub Pawlak, Agnieszka Olma, Mirosław Nęcki, Anna Pióro-Lewandowska, Małgorzata Węcławek, Igor Gumennyi, Piotr Pasek, Witold Bratkowski, Mariusz Śledź, Piotr Przybyłowski, Tomasz Hrapkowicz, Maciej Urlik

**Affiliations:** 1 Department of Cardiac, Vascular and Endovascular Surgery and Transplantology, Silesian Center for Heart Diseases in Zabrze, Medical University of Silesia, 40-055 Katowice, Poland; tstacel@tlen.pl (T.S.);; 2 Student’s Scientific Society, Department of Cardiac Surgery, Transplantology, Vascular and Endovascular Surgery, School of Medical Sciences in Zabrze, Medical University of Silesia, 40-055 Katowice, Poland; 3 Department of Interstitial Lung Diseases and Transplantology, The Saint Paul II Hospital, 31-202 Krakow, Poland; 4 Centre for Transplantology and Interstitial Lung Diseases, Jagiellonian University, 31-202 Krakow, Poland; 5 Department of Cardiac Anaesthesia and Intensive Care, Silesian Centre for Heart Diseases in Zabrze, Medical University of Silesia, 40-055 Katowice, Poland; 6 Department of Cardiothoracic and Vascular Surgery, Memorial Hermann Heart and Vascular Institute, Texas Medical Center, Houston, TX 77030, USA; 7 Health Science Centre at Houston, The University of Texas, Houston, TX 77030, USA

**Keywords:** Birt–Hogg–Dubé syndrome, Foliculin gene mutation, pulmonary cysts, spontaneous pneumothorax, lung transplantation

## Abstract

Birt–Hogg–Dubé Syndrome (BHDS) is a rare autosomal dominant disorder associated with mutations in the Foliculin gene (FLCN), characterized by skin lesions, renal tumors (often malignant or leading to malignancy), and lung cysts, often leading to spontaneous pneumothorax. Lung function is generally preserved in these patients, which is why lung transplantation (LTx) has not been performed in this population to date. Below, we present the case of a 15-year-old boy with BHDS, with extensive cystic lung disease, complicated by recurrent pneumothorax (requiring pleurodesis) and, in his case, progressive respiratory failure, who ultimately underwent double sequential lung transplantation (DLTx). Postoperative management included treatment of humoral rejection and treatment of SARS-CoV-2 infection, in addition to typical immunosuppression. To our knowledge, this is the first DLTx for BHDS reported worldwide. The patient underwent a double lung transplant due to a life-threatening recurrent pneumothorax. This case highlights the importance of multidisciplinary care and demonstrates the feasibility of transplantation in advanced cases of BHDS (despite prior pleurodesis).

## 1. Introduction

BHDS, first described in 1977 by Birt, Hogg, and Dubé, is a rare autosomal dominant disorder associated with mutations in the FLCN tumor suppressor gene encoding folliculin [1,2,3]. Clinically, BHDS is characterized by multiple pulmonary cysts, recurrent spontaneous pneumothorax, fibronodular skin tumors, and renal tumors of variable histology [3,4,5,6,7,8,9,10]. Diagnosis is usually made between the third and fourth decades of life and is not related to gender. The basic diagnostic criteria for BHDS include the presence of skin lesions and FLCN gene mutations [2,3,11].

The exact role of folliculin is not fully understood, but it is thought to act as a tumor suppressor [3,6,11]. In contrast to other cystic lung diseases, BHDS is not usually associated with progressive decline of lung function or chronic respiratory failure [1].

## 2. Detailed Case Description

**Figure 1:**
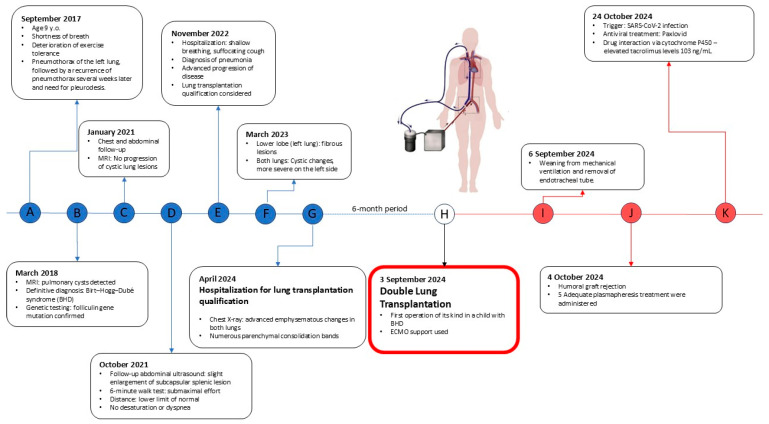
Life span of the child with Birt–Hogg–Dubé syndrome with important events. (A–G)—letters highlighted in blue—represent the pre-transplantation period. (I–K)—letters highlighted in red—represent the post-transplantation period. (H)—remains uncoloured indicating the day of the surgery.

We present a case of a 15-year-old male patient with a significant medical history of cystic lung disease and recurrent pneumothorax who was admitted to our lung transplant department for evaluation for DLTx (Figure 1).

The patient was diagnosed with lung sequestration, extensive cystic lesions in the lungs, and recurrent left- and right-side pneumothoraces. He had to undergo pleurodesis for the pneumothoraces. Talc pleurodesis slurry via the chest tubes was performed. That adhesions were formed locally, and therefore this technique means that not always the entire pleural cavity is occupied by adhesions. In addition, allergic rhinitis was found, and a subcapsular splenic lesion was observed. The patient reported episodic shortness of breath and vomiting, accompanied by transient deterioration of physical performance. Oxygen saturation during sleep and rest showed no decrease below 93%. Exercise tests didn’t reveal significant desaturation.

Interventional radiology was consulted regarding the splenic lesion, which was deemed likely to be benign and vascular in nature, without the need for tissue sampling.

**Figure 2:**
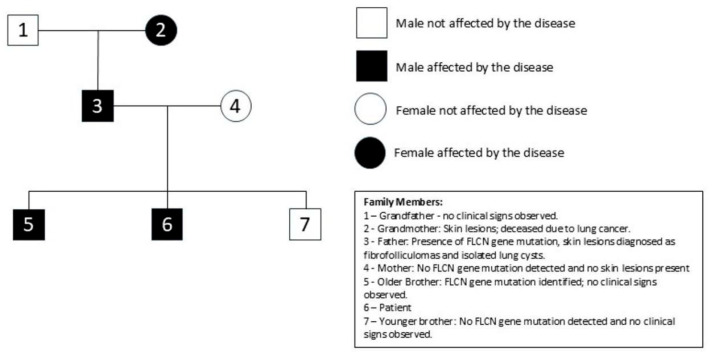
Family history of the disease.

Family history was positive for lung cancer and skin lesions on the father’s side. Genetic testing confirmed a pathogenic FLCN mutation inherited from a father in whom fibrofollicular facial tumors and isolated lung cysts were observed (Figure 2).

**Figure 3:**
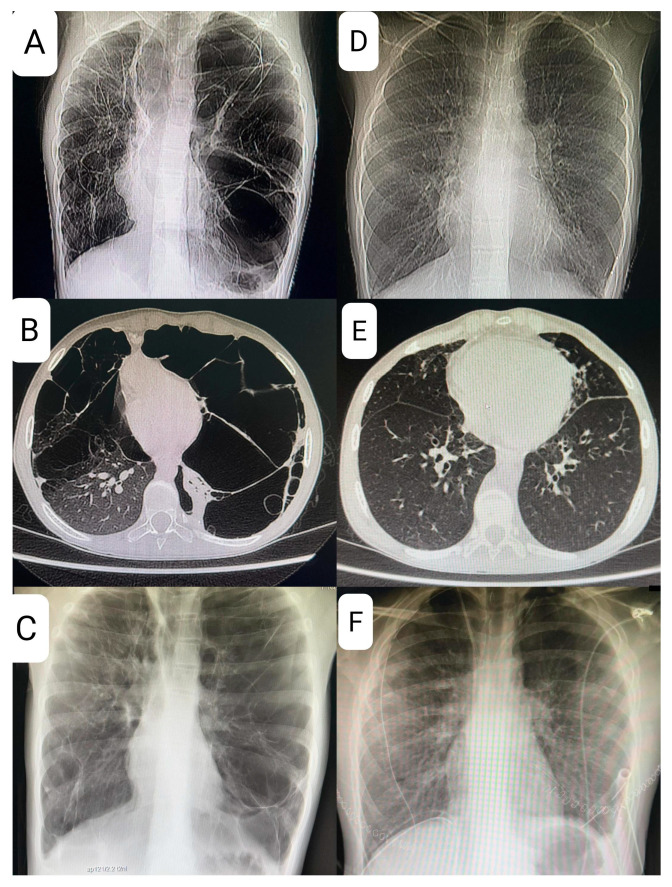
(**A**–**C**)—preoperative images—large cysts and shift of the mediastinum to the right; (**A**): CT topogram, (**B**): CT transverse section, (**C**): X-ray p–a; (**D**–**F**)—postoperative images—normal lung tissue; (**D**): CT topogram, (**E**): CT transverse section, (**F**): X-ray.

At age 9, the patient presented with shortness of breath and decreased exercise tolerance, leading to a diagnosis of left-sided pneumothorax, followed by a recurrence of pneumothorax several weeks later and the need for pleurodesis. Magnetic resonance imaging (MRI), X-ray, and computed tomography (CT) of the chest revealed multiple lung cysts, and BHDS was confirmed by genetic testing (Figure 3).

In the following years, the child reported intermittent shortness of breath and chest pain, but exercise capacity remained moderate. Follow-up imaging in 2021 did not demonstrate progression of lung lesions, although slight enlargement of the splenic lesion was noted, requiring further close monitoring. The patient underwent diagnostic imaging, which included a computed tomography scan for kidney tumors. Additionally, a general urine test did not reveal any abnormalities.

In November 2022, the patient was hospitalized for shortness of breath and cough due to pneumonia. Imaging studies showed parenchymal-interstitial infiltrates and progression of cystic changes, with a rightward mediastinal shift.

The patient’s lung disease was considered to be severe, and given the lack of effective pharmacotherapy, the patient was considered for LTx after all contraindications were excluded. Reevaluation in early 2024 confirmed advanced cystic degeneration with extensive bullae, areas of fibrous thickening, and reduced vascular architecture—all of these findings were considered life-threatening. A 6 min walk test showed significant exercise desaturation, which, together with findings mentioned above, prompted placement on the list for DLTx.

In September 2024, a suitable donor (Donor after brain death determination—BDD, 37 years old) became available. The patient underwent DLTx using venoarterial extracorporeal membrane oxygenation (ECMO) (via the right groin vessel access). The use of venoarterial ECMO was necessary for two reasons: pulmonary hypertension caused by the course of the disease (WHO 3) and because of the small amount of functional lung tissue, which prevented efficient single-lung ventilation during sequential LTx. First, a left thoracotomy was performed, and then, after dissection of the lung hilum elements, a left lung resection was performed. Due to talc pleurodesis, preparation was difficult in places with the largest adhesions, where even extrapleural preparation had to be performed. Bronchial and vascular anastomoses were created, after which ventilation and perfusion were restored. Similarly, resection and implantation were performed on the right side. Intraoperative bronchoscopy confirmed good quality and adequate patency of the anastomoses, and due to the hemodynamically efficient heart function, ECMO was successfully weaned and disconnected while still in the operating room. The authors performed the procedure using thoracotomy access due to the fact that this technique brings less pain to the patient, allows for faster rehabilitation, and provides a better cosmetic effect. At the center where the operation was performed, over 98% of procedures are performed using thoracotomy access, so it was decided to perform the operation using this method.

After the operation, the patient was ventilated in BIPAP mode. Sedation was appropriately weaned, and extubation was achieved after 74 h. Diuresis remained stable, and chest radiography did not show any signs of primary graft dysfunction. Low-flow oxygen via nasal cannula was necessary for several hours after extubation.

On hospital day 28, routine LUMINEX testing revealed the presence of donor-specific anti-HLA antibodies against antigens A2, DQ7, and DQ8 in the amount of over 2000 Mean Fluorescence Intensity (MFI), suggesting humoral rejection. According to this finding, patient was treated with five plasmapheresis sessions and received a rituximab infusion with good tolerance and normalization of antibody levels.

During hospitalization, tacrolimus levels were unstable due to concurrent SARS-CoV-2 infection treated with Paxlovid, which interacts with cytochrome P450 enzymes, and stabilized after dose adjustments. Pulmonary function tests, however, showed an FEV1 > 80% of predicted, and imaging studies did not show any evidence of graft dysfunction or chronic rejection. The patient achieved correct multiorgan function, stable oxygen saturation (>95%) and was discharged home in good general condition after 53 days of hospitalization.

## 3. Discussion

Birt–Hogg–Dubé syndrome typically presents with renal tumors, benign skin lesions, and pulmonary cysts. Skin and pulmonary lesions are more common than renal tumors [6]. BHDS, associated with mutations in the FLCN gene, shows the presence of pulmonary cysts, a propensity for spontaneous pneumothoraces, and an increased risk of renal neoplasms and skin lesions [5]. In a high percentage of cases, pulmonary lesions do not lead to end-stage respiratory failure, which indicates and explains the rarity of qualification for lung transplantation [2,12].

BHDS is associated with multiple, bilateral, thin-walled lung cysts in 80–90% of patients, usually located in the basal and mediastinal regions, occurring characteristically subpleuraly. Lung function in BHDS is generally preserved or shows only mild impairment, and respiratory failure is rare, distinguishing it from other cystic lung diseases (lymphangioleiomyomatosis (LAM) and Pulmonary Langerhans Cell Histiocytosis (PLCH) [2,5,6,7,8,12,13,14,15]. Studies have consistently shown that most patients with BHDS maintain normal or near-normal spirometry and diffusing capacity of the lung for carbon monoxide (DLCO) [13,15].

There is a hypothesis suggesting that cyst formation may arise from impaired cell–cell adhesion and repeated stretch-induced stress caused by breathing. Over time, this leads to the expansion of alveolar spaces. It is seen especially in lung regions where alveolar volume changes throughout the respiratory cycle [3,4,8,12]. This hypothesis delivers an explanation for why BHDS-associated lung cysts predominantly affect the basilar and mediastinal areas of the lung in contrast to other cystic lung diseases, where cysts are multifocally located or present as diffuse changes [3,7,8,12,13].

The hallmark of BHDS on high resolution computed tomography (HRCT) is the presence of cysts with irregular shapes and varying sizes and numbers, with the surrounding normal lung parenchyma remaining intact. However, the mere presence of these cysts is insufficient for a confident diagnosis. In our case, a CT scan showed significant progression of cystic changes in the lungs with displacement of the heart. In patients with BHDS, the number of cysts ranges from a few to about 400, but most patients have fewer than 20 cysts and occupy less than 30% of the lung volume [6]. Cyst expansion and rupture have been documented with changes in atmospheric pressure, such as flying [5]. Since cystic lung disease rarely affects lung function in BHDS, regular follow-up with pulmonary function tests is not recommended. However, patients with cystic lung disease who have impaired lung function should have periodic testing from the beginning of diagnosis. As of today, there is no specific treatment for BHDS-associated cystic lung disease and no reports on the efficacy of mTOR inhibitor treatment [7]. In Birt–Hogg–Dubé syndrome, inactivation of the FLCN tumor suppressor gene leads to dysregulation of the mTOR signaling pathway, which plays a central role in the control of cell proliferation and survival, promoting neoplastic transformation in renal tissue and the development of pulmonary cysts. This has positioned the mTOR axis as a possible therapeutic strategy in BHDS.

Pneumothorax is often the initial clinical manifestation of BHDS, with a prevalence of 30% to 60%, and is 30–50 times more frequent than in the general population. The mean age of occurrence of the first episode of pneumothorax is between 20 and 40 years [3,5,6,7,12,13,16,17]. The presence of pulmonary cysts, along with their number, size, and cumulative volume, and a positive family history of pneumothorax, has been recognized as a risk factor for the occurrence of pneumothorax in BHDS. The risk of pneumothorax is greatly increased by larger cyst size and basal distribution. Spontaneous pneumothorax is classified as an emergency condition manifesting as dyspnea and chest tightness. Secondary spontaneous pneumothorax is most common in cystic fibrosis and chronic obstructive pulmonary disease, and less common in tuberculosis, primary lymphangioleiomyomatosis, Langerhans cell histiocytosis, and BHDS [5]. Both pneumothorax and, in particular, its recurrence are recognized as life-threatening risk factors, especially when associated with respiratory failure; therefore, at this stage of the disease, lung transplantation should be considered. Disease severity increases with age but remains unrelated to smoking exposure [2,16]. It is worth considering whether BHDS should be suspected in all patients presenting with spontaneous pneumothorax, as such an approach would increase vigilance for the possible development of renal cancer, which is common in this condition [16].

Characteristic symptoms in BHDS include skin lesions in the form of fibrofolliculomas and trichodiscomas, with an 80–90% probability of occurrence. In histopathology, they are characterized by bands of proliferating epithelial cells surrounded by a thick layer of connective tissue [2]. Skin lesion numbers increase with age [6,7,12]. Fibrofolliculomas and trichodiscomas most commonly appear on the face, neck, and trunk and usually develop between 35 and 45 years of age but may also be absent [2,6,7,17,18,19]. Therapeutic options are limited to laser ablation, shave and cautery treatment, curettage, and excision [19].

Renal lesions most often present as hybrid oncocytic renal cell carcinomas. They account for half of all renal tumor diagnoses in BHDS [2]. The lifetime risk of renal cancer ranges from 14 to 35%, and the average age of onset is 46–52 years [6,18]. The risk of kidney cancer is 7 times higher in BHDS patients compared to their unaffected siblings. Family members of BHDS patients who have symptoms of this syndrome are also at greater risk of developing kidney cancer than other members of the general population [6]. In these patients, chromophobe renal cell carcinoma, clear cell renal cell carcinoma, or oncocytoma are also observed with a frequency of 33%, 9%, and 5%, respectively. Therefore, such patients should start screening for kidney cancer, and as USG examination is less likely to detect small lesions, MRI and CT are the preferred tools. The screening should start at the age of 20 and be continued every 3 years [3,6].

Treatment of pneumothorax in BHDS varies with stage. While conservative measures may be sufficient for isolated events, recurrent pneumothorax often requires surgical intervention, including video-assisted thoracoscopy (VATS), bullectomy, and pleurodesis [7,20,21]. In a case study published in 2017, Takegahara and colleagues described a patient with recurrent pneumothoraces. Thoracoscopic bilateral bullectomy with curative intent was performed with a finding of extremely numerous cysts measuring 2–5 mm in the lung apex, interlobular region, and mediastinum. To prevent recurrence, total pleural covering was performed, using polyglycolic acid sheets and administered fibrinogen solution containing factor XIII. This method, although more advanced, appears to be more effective in the long-term prevention of recurrence compared to traditional pleurodesis [20]. However, pleurodesis may complicate future lung transplantation because of pleural adhesions and increased surgical risk of severe hemorrhage, sometimes even making the operation impossible. In our case, extensive bilateral pleurodesis posed a significant surgical challenge but did not preclude successful transplantation.

In another case, after VATS on the right lung with cyst resection and ablation, the patient returned a month later with a left-sided pneumothorax. A similar surgical procedure was performed on the left lung. At the six-month follow-up after the second operation, no episodes of pneumothorax were detected in either lung [13,18]. Intraoperative use of excessive positive pressure ventilation should be avoided to minimize the risk of a cyst rupture leading to a possible intra- and postoperative pneumothorax [3]. Additionally, unnecessary intraoperative sealing tests should be avoided to minimize the risk of rupture in thin-walled cysts [20].

BHDS is presumed to be underdiagnosed due to its significant phenotypic variability and incomplete penetrance. It is recommended that both symptomatic and asymptomatic individuals begin regular renal cancer screening at the age of 20 years old and receive expert care to help prevent recurrence of spontaneous pneumothorax [22]. In cases where this syndrome or other forms of polycystic lung disease are suspected, physicians are advised to consider total pleural coverage, but with the caveat that pleurodesis may preclude (due to technical reasons and the significant risk of bleeding) future lung transplantation [20].

An additional consideration in screening pertains to whether chest CT should be performed in patients diagnosed with Birt–Hogg–Dubé syndrome based on dermatological or renal findings. In individuals with a confirmed diagnosis of BHDS, high-resolution chest CT is recommended, as studies show that 80–100% of patients with the disease have pulmonary cysts [21]. Children with spontaneous pneumothorax are not routinely subjected to chest CT, which often reveals subpleural basal lung cysts—key indicators for diagnosis that often remain undetected in other sources. While chest CT is not recommended as a standard diagnostic approach in pediatric or adolescent pneumothorax, it is crucial to enhance awareness of Birt–Hogg–Dubé syndrome among specialists in relevant fields [22].

Relatives of affected individuals should undergo appropriate screening for lung and kidney involvement. Due to the fact that frequent occurrences of pulmonary cysts and kidney tumors are highly prevalent in BHDS, genetic testing, either diagnostic or presymptomatic, should be considered [7].

For the diagnosis and recognition of this disease entity, the literature provides relevant criteria worth remembering. They are divided into two groups: primary and secondary. Diagnosis requires one primary criterion or two secondary criteria. The first primary criterion involves at least five fibrofolliculomas or trichodiscomas, with at least one of them confirmed histologically. The second major criterion is a pathogenic FLCN germline mutation. Detection of mutations in the FLCN gene allowed confirmation of the diagnosis of BHDS in the patient presented in this case. It is noteworthy that clinical manifestations can be subtle or incomplete. Our patient met numerous minor criteria while meeting only one of the main criteria—FLCN gene mutations. 40% of patients meet the criteria for diagnosis, despite negative FLCN gene testing. For this reason, the presence of only a negative FLCN gene testing result does not mandate rejection of the diagnosis of BHDS. There are three minor criteria: multiple lung cysts, which are bilateral and basally located; renal cancer, which is diagnosed before the age of 50 years old; and a first-degree relative with BHDS. Our patient has two relatives—a father and a brother with—confirmed diagnoses of BHDS and multiple lung cysts [23].

## 4. Conclusions

To our knowledge, this is the first reported case of bilateral lung transplantation in a patient with BHDS. This case demonstrates that LTx is feasible in selected pediatric patients with BHDS and advanced lung disease, despite previous pleurodesis and complex airway pathology. It highlights the importance of early diagnosis, multidisciplinary treatment, and an individualized therapeutic approach in this rare and challenging disease.

## Data Availability

The anonymized dataset supporting the findings of this study is openly available upon reasonable request to the corresponding author. In accordance with institutional and ethical guidelines, the dataset has been deidentified to ensure patient confidentiality. We fully support the principles of open data and transparency in scientific research. Therefore, researchers interested in replicating or expanding upon our analyses are welcome to request access to the data, which will be shared in compliance with ethical regulations and applicable data use agreements. For access to the dataset, please contact authors Tomasz Stacel and Jakub Pawlak.

## Abbreviations

The following abbreviations are used in this manuscript:

BHDS	Birt–Hogg–Dubé syndrome
BDD	Brain death determination
FLCN	Folliculin gene
LTx	Lung transplantation
DLTx	Double sequential lung transplantation
LAM	Lymphangioleiomyomatosis
PLCH	Pulmonary Langerhans cell histiocytosis
VATS	Video-assisted thoracoscopy
MRI	Magnetic resonance imaging
CT	Computed tomography
ECMO	Extracorporeal membrane oxygenation
HRCT	High resolution computed tomography
MFI	Mean fluorescence intensity
DLCO	Diffusing capacity of the lung for carbon monoxide
